# Supplementary material for: Multi‐dataset identification of innovative feature genes and molecular mechanisms in keratoconus
Source: J Cell Mol Med. 2024 Sep 19;28(18):e70079. doi: 10.1111/jcmm.70079 (PMC11412914; doi:10.1111/jcmm.70079)
Supplement: Supplementary file 2 — Material S2. [file JCMM-28-e70079-s002.zip › 7.GSEA analysis/1.ARL11/KEGG_ASCORBATE_AND_ALDARATE_METABOLISM.html]

Details for gene set KEGG\_ASCORBATE\_AND\_ALDARATE\_METABOLISM[GSEA]

|  || Dataset | ARL11.group.cls#h\_versus\_l |
| Phenotype | group.cls#h\_versus\_l |
| Upregulated in class | h |
| GeneSet | KEGG\_ASCORBATE\_AND\_ALDARATE\_METABOLISM |
| Enrichment Score (ES) | 0.55760187 |
| Normalized Enrichment Score (NES) | 1.5146574 |
| Nominal p-value | 0.01632653 |
| FDR q-value | 0.7817075 |
| FWER p-Value | 0.774 |
Table: GSEA Results Summary

  

Fig 1: Enrichment plot: KEGG\_ASCORBATE\_AND\_ALDARATE\_METABOLISM      
 Profile of the Running ES Score & Positions of GeneSet Members on the Rank Ordered List

  

| SYMBOL | TITLE | RANK IN GENE LIST | RANK METRIC SCORE | RUNNING ES | CORE ENRICHMENT || 1 | UGT2B7 | na | 1178 | 0.368 | 0.1319 | Yes |
| 2 | UGT2B15 | na | 2914 | 0.244 | 0.1921 | Yes |
| 3 | UGT2B11 | na | 2923 | 0.244 | 0.3020 | Yes |
| 4 | UGT2B4 | na | 3576 | 0.216 | 0.3807 | Yes |
| 5 | UGT1A8 | na | 3762 | 0.209 | 0.4697 | Yes |
| 6 | UGT1A6 | na | 4803 | 0.172 | 0.5173 | Yes |
| 7 | UGT2B28 | na | 5683 | 0.146 | 0.5576 | Yes |
| 8 | UGT2B10 | na | 9893 | 0.062 | 0.4641 | No |
| 9 | ALDH7A1 | na | 10913 | 0.048 | 0.4566 | No |
| 10 | ALDH3A2 | na | 13283 | 0.025 | 0.3994 | No |
| 11 | UGT2A3 | na | 13634 | 0.022 | 0.3991 | No |
| 12 | UGDH | na | 19859 | -0.023 | 0.2301 | No |
| 13 | MIOX | na | 20646 | -0.029 | 0.2204 | No |
| 14 | UGT2A1 | na | 20689 | -0.029 | 0.2322 | No |
| 15 | ALDH1B1 | na | 25126 | -0.078 | 0.1394 | No |
| 16 | ALDH9A1 | na | 25157 | -0.078 | 0.1738 | No |
| 17 | ALDH2 | na | 31150 | -0.226 | 0.1031 | No |
Table: GSEA details [plain text format]

  

Fig 2: KEGG\_ASCORBATE\_AND\_ALDARATE\_METABOLISM      
 Blue-Pink O' Gram in the Space of the Analyzed GeneSet

  

Fig 3: KEGG\_ASCORBATE\_AND\_ALDARATE\_METABOLISM: Random ES distribution      
 Gene set null distribution of ES for **KEGG\_ASCORBATE\_AND\_ALDARATE\_METABOLISM**
